# Supplementary material for: Metal to phosphorus stoichiometries for freshwater phytoplankton in three remote lakes
Source: PeerJ. 2016 Dec 20;4:e2749. doi: 10.7717/peerj.2749 (PMC5178342; doi:10.7717/peerj.2749)
Supplement: Table S1 — Fully quantitative concentrations of metals and P that showed linearity in the calibration curves computed by Plasmalab. These were subsequently used in the regression analysis to determine the concentration of the elements in the unknown sample solutions. [file peerj-04-2749-s001.docx]

**Supplemental Table S1.** Fully quantitative concentrations of metals and P that showed linearity in the calibration curves computed by Plasmalab. These were subsequently used in the regression analysis to determine the concentration of the elements in the unknown sample solutions.

**Standard concentrations used for calibration (*µ*g/l)**

| **Std.** |  |  |  |  |  |  |  |
| --- | --- | --- | --- | --- | --- | --- | --- |
| **label** | **Na** | **Mg** | **P** | **Cr** | **Mn** | **Fe** | **Co** |
| **1**  **2**  **3**  **4**  **5**  **6** | \|  \| \| --- \| \|  \| \|  \| \| 100.0 \| \| 1000.0 \| \| 5000.0 \| | \|  \| \| --- \| \| 1.0 \| \| 10.0 \| \| 100.0 \| \| 1000.0 \| \|  \| | \|  \| \| --- \| \|  \| \|  \| \| 100.0 \| \| 1000.0 \| \|  \| | \|  \| \| --- \| \| 1.0 \| \| 10.0 \| \| 100.0 \| \| 1000.0 \| \|  \| | \|  \| \| --- \| \| 1.0 \| \| 10.0 \| \| 100.0 \| \|  \| \|  \| | \|  \| \| --- \| \|  \| \|  \| \| 100.0 \| \| 1000.0 \| \|  \| | \| 0.1 \| \| --- \| \| 1.0 \| \| 10.0 \| \| 100.0 \| \| 1000.0 \| \|  \| |
|  | **Ni** | **Cu** | **Zn** | **Cd** | **Hg** | **Pb** |  |
| **1**  **2**  **3**  **4**  **5**  **6** | \| 0.1 \| \| --- \| \| 1.0 \| \| 10.0 \| \| 100.0 \| \| 1000.0 \| \|  \| | \| 0.1 \| \| --- \| \| 1.0 \| \| 10.0 \| \| 100.0 \| \| 1000.0 \| \|  \| | \| 0.1 \| \| --- \| \| 1.0 \| \| 10.0 \| \| 100.0 \| \| 1000.0 \| \|  \| | \| 0.1 \| \| --- \| \| 1.0 \| \| 10.0 \| \| 100.0 \| \| 1000.0 \| \|  \| | \| 0.1 \| \| --- \| \| 1.0 \| \| 10.0 \| \| 100.0 \| \|  \| \|  \| | \| 0.1 \| \| --- \| \| 1.0 \| \| 10.0 \| \| 100.0 \| \|  \| \|  \| |  |
